# Emerging Roles of G Protein-Coupled Receptors in Hepatocellular Carcinoma

**DOI:** 10.3390/ijms19051366

**Published:** 2018-05-04

**Authors:** Wen-Ting Peng, Wu-Yi Sun, Xin-Ran Li, Jia-Chang Sun, Jia-Jia Du, Wei Wei

**Affiliations:** 1 Institute of Clinical Pharmacology, Anhui Medical University, Hefei 230032, China; Wendy765138970@126.com (W.-T.P.); m15555430882@163.com (X.-R.L.); sunjc5678@163.com (J.-C.S.); 18375315827@163.com (J.-J.D.); wwei@ahmu.edu.cn (W.W.); 2 Key Laboratory of Antiinflammatory and Immune Medicine, Ministry of Education, Hefei 230032, China; 3 Anhui Collaborative Innovation Center of Anti-Inflammatory and Immune Medicine, Hefei 230032, China

**Keywords:** G protein coupled receptor, hepatocellular carcinoma, chemokines, lysophosphatidic acid, adrenergic receptor, signal transduction

## Abstract

Among a great variety of cell surface receptors, the largest superfamily is G protein-coupled receptors (GPCRs), also known as seven-transmembrane domain receptors. GPCRs can modulate diverse signal-transduction pathways through G protein-dependent or independent pathways which involve β-arrestins, G protein receptor kinases (GRKs), ion channels, or Src kinases under physiological and pathological conditions. Recent studies have revealed the crucial role of GPCRs in the tumorigenesis and the development of cancer metastasis. We will sum up the functions of GPCRs—particularly those coupled to chemokines, prostaglandin, lysophosphatidic acid, endothelin, catecholamine, and angiotensin—in the proliferation, invasion, metastasis, and angiogenesis of hepatoma cells and the development of hepatocellular carcinoma (HCC) in this review. We also highlight the potential avenues of GPCR-based therapeutics for HCC.

## 1. Introduction

Hepatocellular carcinoma (HCC) is the second most prevalent cause of mortality and poor-prognosis malignancy characterized by recurrence after surgery and metastasis worldwide [1]. While surgery with either resection or orthotopic liver transplantation is the only effective curative treatment, many HCC patients are diagnosed at an advanced stage at which surgical therapies are futile [2]. Recent studies have revealed the crucial role of G protein-coupled receptors (GPCRs) in tumorigenesis and the development of HCC. GPCRs make up the largest superfamily of signal transduction proteins, playing multiple biological roles through coupling to a heterotrimeric G protein contacted with the inner surface of the plasma membrane. Nevertheless, aberrant expression of GPCRs alter normal physiological signaling pathways and govern the proliferation, survival, angiogenesis, invasion, migration, metastasis, metabolism, or immune response in HCC initiation and progression. Furthermore, oncogenes often cooperate with altered signaling nodes (onco-modulators) to strengthen tumoral hallmarks and lead to cancer development. As a result, there is an ideal opportunity for the development of novel, mechanism-based strategies for diagnosis, prevention, and treatment of HCC through interfering with GPCRs and their downstream signal targets.

## 2. Structure and Signal Transduction of G Protein-Coupled Receptors (GPCRs)

### 2.1. Structure and Classification of GPCRs

GPCRs, which are vitally important eukaryotic signal transduction transmembrane proteins, constitute the largest protein family, with over 800 members in the proteome of humans. They have a common structure of seven transmembrane helices (H1–H7) joined with three intracellular (I1, I2 and I3) and three extracellular (E1, E2 and E3) loops, an extracellular N-term, and an intracellular C-term. In addition, according to sequence and structure similarity, GPCRs were usually grouped into six main families: class A (rhodopsin-like), class B (secretin receptor family), class C (metabotropic glutamate/pheromone), class D (fungal mating pheromone receptors), class E (cyclic adenosine monophosphate (cAMP) receptors), and class F (frizzled/smoothened) [3]. Class A is the largest and most extensively studied. It is used in the binding of small peptides and biogenic amines. Class B includes 15 peptide hormones receptors, which can be classified into five subfamilies on the basis of their physiological function. Class C encompasses receptors for three taste type 1 receptors, the calcium-sensing and γ-aminobutyric acid type B (GABA_B_) receptors, a class of eight metabotropic glutamate receptors, and a number of orphan receptors. Class D chemically communicates in some of the organisms. Class E is used to form the chemotactic signaling system of slime molds. Finally, class F is essential for Wnt binding [4].

### 2.2. Signal Transduction of GPCRs

The classical pathway through which GPCRs mediate cellular physiology is that the bundle with ligands induces conformational alteration in the transmembrane and intracellular domains of the receptor, thus communicating with heterotrimeric G proteins that consist of the Gα, Gβ, and Gγ subunits. Activated GPCRs catalyze the liberation of guanosine diphosphate (GDP) and then bundle with guanosine triphosphate (GTP) for G protein activation. Afterwards, both the Gα-GTP and Gβγ subunits lead to various signaling cascades and different downstream effector proteins to regulate large quantities of cellular physiology [5].

GPCRs transduce signals through their direct interactions with β-arrestins. GPCRs scaffold many intracellular signaling proteins by different signaling pathways and induce signaling cascades independent of G protein activation—including Wnt, Notch, transforming growth factor-β (TGF-β), and the hedgehog (Hh) pathways—as well as downstream kinases—such as phosphoinositide 3-kinase (PI3K) and mitogen-activated protein kinase (MAPK) [6]. Additionally, activated GPCRs also interact with GRKs or other receptors, mediating the downstream signal pathway [7].

Further, GPCRs can utilize receptor tyrosine kinases (RTKs) to mediate important cellular responses, such as proliferation, differentiation, and survival [8]. Depending on the receptor and cell type, GPCRs signaling involves transactivation of several different RTKs, such as insulin-like growth factor 1 receptor (IGF1R), epidermal growth factor receptor (EGFR), and platelet-derived growth factor receptor (PDGFR) [9,10,11]. Then, numerous growth factors use G proteins and associated signaling molecules that participate downstream of RTKs to signal to effectors. For instance, FPR2 (a GPCR) induces the phosphorylation of Y1313/Y1349/Y1356 residues of c-Met and triggers some of the molecular responses elicited by c-Met/HGF binding, such as STAT3, PLC-γ1/PKCα, and PI3K/Akt pathways [12].

## 3. Role of GPCRs in the Occurrence and Development of Hepatocellular Carcinoma (HCC)

**Figure 1:**
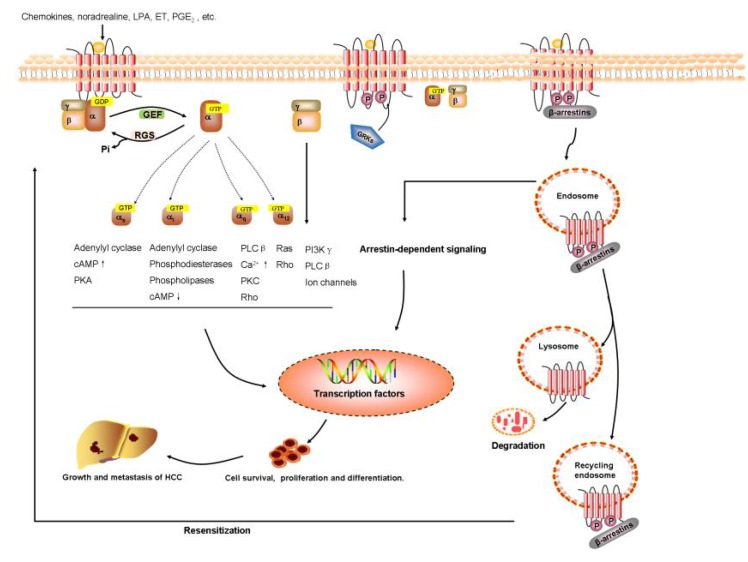
GPCRs signaling pathway in HCC. The aberrant expression of GPCRs alter normal physiological signaling pathways, and govern the proliferation, survival, angiogenesis, invasion, migration, metastasis, metabolism, or immune response in HCC initiation and progression.

Increasing clinical and experimental data have suggested that GPCRs play a vital but not fully understood role in HCC progression and metastasis by modulating diverse downstream signaling pathways. Furthermore, we have learned that GPCRs—particularly those coupled to chemokines, prostaglandin, lysophosphatidic acid, endothelin, catecholamine, and angiotensin—have a central function in proliferation, invasion, metastasis, and neovascularization of hepatoma cell and the development of HCC, which are summarized in Figure 1. Therefore, these new findings might provide potential avenues for the development of novel, GPCR-based therapeutics strategies for cancer diagnosis, prevention, and treatment.

### 3.1. Chemokine Receptors

Chemokines, which are small proinflammatory chemoattractant cytokines, couple to GPCRs to induce conformational alteration and trigger intracellular signaling pathways containing cell movement and activation [13]. To date, there are 20 identified chemokine receptors, which are grouped into four subfamilies according to the chemokine ligands to which they couple. Therefore, receptors that couple to C-, CC-, CXC-, and CX3C-chemokines are named XCRn, CCRn, CXCRn, and CX3CRn, respectively [14]. The chemokine system not only acts as the central element in regulating normal immune response, it also plays an important role in the microenvironment of HCC. There, the HCC cells express chemokine receptors that bind to chemokines to directly regulate the functions of tumor cells, including proliferation, migration, invasion, and apoptosis. Likewise, the chemokine system plays multiple roles in immune cells and other stroma cells during the development of HCC. Several studies have demonstrated that PI3K/Akt was one of the key pathways under chemokine receptors that has a crucial effect in the occurrence and development of HCC [15,16,17,18].

#### 3.1.1. CCRn

The CC subclass is the largest group in all chemokine receptors that has a significant influence on HCC. CCR1 is aberrantly overexpressed in human HCC tissues, *N*-nitrosodiethylamine (DEN)-induced HCC, and HCC induced by hepatitis B virus surface (HBs) antigen-primed splenocyte transfer to myelo-ablated syngeneic HBs antigen transgenic mice. CCL2, CCL3, and CCL15—cognate ligands of CCR1—markedly increase in endothelial cells from HCC tissues. Furthermore, proliferation of endothelial cells also significantly enhances and directly modulates vessel remodeling via bundling with CCR1 in an autocrine way [19,20]. Further studies find that tumor angiogenesis strikingly diminishes in CCR1-deficient mice. A rich source of growth factors, intratumoral Kupffer cells and matrix metalloproteinases (MMPs) also decrease in CCR1-deficient HCC mice [21]. Furthermore, CCR1 knockdown results in the reduction of migration, invasion, and pulmonary metastasis in vitro and in vivo through inhibiting PI3K/Akt and hypoxia-inducible factor 1 (HIF-1)α in HCC cells [15]. CD11b/Gr1mid (a myeloid cell subset) cells are recruited from bone marrow by tumor-derived CCL2 and they increase in number markedly during development of liver metastases. Depletion of the CD11b/Gr1mid in a transgenic CD11b-diphtheria toxin receptor mouse model dramatically decreases tumor-cell proliferative ability. Also, recruitment of CD11b/Gr1mid cells depends upon CCL2/CCR2 and its depletion significantly decreases tumor burden [22]. In addition, the downregulated microvessel density is associated with a reduced number of macrophages and hepatic stellate cells (HSC) that are core members during neovascularization in the CCR2-deficient mice [23].

CCR5 and its ligand CCL3, which regulates inflammation, are crucial in the microenvironment of HCC, recruiting packs of macrophages and neutrophils into the inflammation sites in different HCC cell lines when stimulated with IL-1α or IL-1β [24].

CCR6 is overexpressed in the intrahepatic metastasis of HCC. Angiogenesis benefits from the accumulation of intratumoral IL-17-producing cells in the microenvironment of HCC. It is due to these cells that CCR6 is highly expressed and a CD45RO^+^CD62L^−^CCR7^−^ effector memory phenotype is shown [25]. However, the role and characteristics of CCR6 in the development of HCC is limited. In addition to IL-17-producing cells, the CCL20/CCR6 axis regulates the migration of circulating Tregs into the tumor microenvironment, which in turn leads to vascular invasion, metastasis, and poor prognosis of HCC patients [26]. Thus, CCR6 might be one of the prognostic factors and a new therapeutic target for HCC.

High expression of CCR7 increases intrahepatic and lymphatic HCC dissemination [27]. However, the functions of the CCL21/CCL19-CCR7 axis in HCC progression remains unknown. In contrast, overexpression of CCL21, either in HCC cells or in dendritic cells, is an effective treatment in HCC models. With the accumulation of CCL21 in tumors, the number of CD4^+^ and CD8^+^ T cells, dendritic cells (DCs), interleukin-12 (IL-12), and interferon-γ (IFN-γ) is dramatically increased. Consequently, microvessels are reduced during the angiogenesis of HCC [28].

CCR9 is strikingly increased in HCC tissue samples. Overexpression of CCR9 is associated with increased tumor nodes, vascular invasion, and a high Edmondson–Steiner grade. Furthermore, ectopic expression of CCR9 increases the proliferative ability and tumorigenicity of HCC cells. However, blocking CCR9 mediates deterioration of HCC cells by decreasing the cell-cycle regulators p21 and p27, as well as increasing cyclin D1 [29]. This study indicates that CCR9 may serve as a potential therapeutic target for HCC.

#### 3.1.2. CXCRn

Another major part of chemokines is composed of the CXC subclass in HCC. Compared with adjacent and normal liver tissues, the level of CXCR2 is significantly enhanced in HCC, which accelerates invasion and metastasis [30]. The CXCL5/CXCR2 axis contributes to the induction of epithelial–mesenchymal transition (EMT) and the migration and invasive ability of HCC cells via activating the PI3K/Akt/GSK-3β/Snail and PI3K/Akt/ERK1/2 signaling pathways [16,17]. CXCL5 also recruits neutrophils into HCC tissues, which favors angiogensis in hepatocarcinogenesis [31]. Taken together, the CXCL5/CXCR2 axis contributes to invasion, migration, and angiogenesis of HCC.

Moreover, the CXCL9/CXCL10-CXCR3 axis has a significant impact on HCC patients’ prognosis. Overexpression of CXCR3 is frequently associated with tumor size, tumor differentiation, portal invasion, and metastasis in HCC tissues via increasing levels of ERK1/2 phosphorylation in the MAPK signaling pathway, then increasing the MMP-2 and MMP-9, and accordingly accelerating invasion and migration of CD133^+^ liver cancer cells [32]. It has been suggested that CXCR3 is assigned to the migration of liver cancer cells.

The angiogenesis, invasion, and tumor growth of human HCC cells are correlated with the overexpression of CXCR4 or CXCR7. Exposure to CXCL12 regulates a perinuclear translocation of CXCR4 in Huh7/Hep3B cells and promotes the invasion of Huh7 cells. Also, the high level of CXCR4 in HCC patients is markedly associated with advanced local tumors, lymphatic metastasis, distant dissemination, and decreased three-year survival rate [33,34,35]. That is, high CXCR4 expression is strikingly correlated with advanced HCC. The CXCL12-CXCR4 axis on HCC cells reorganizes the cytoskeleton and increases MMP-9 and MMP-2, both of which upregulate migration and invasion [36]. Highly expressed CXCR4 contributes to the increased migration and invasion of HCC cell in the EMT system, and some studies suggest that CXCR4 accumulates at the perivascular region and tumor border [37]. Next, CXCL12 comes from HSC inducing EMT and the enhancement of migration in HCC cells [38]. Furthermore, CXCR7—the other receptor for CXCL12—is increased in highly invasive cell lines and HCC tumor tissues. CXCR7 plays a vital role in the proliferation, migration, invasion, and angiogenesis of HCC cells via activation of MAPK and upregulation of vascular endothelial growth factor (VEGF)-A and galectin-3, which promote tumor invasion and angiogenesis. Silencing CXCR7 by small interfering RNAs (siRNAs) results in reduced proliferation, migration, invasion, adhesion, and angiogenesis abilities, which are partially due to the downregulation of MMP-2 and MMP-9 [39,40,41]. However, it has been confirmed that the expression of CXCR7 is upregulated on endothelial cells, but neither on human primary hepatocytes nor HCC cell lines in a large amount of 408 HCC tissues. Furthermore, the level of CXCR7 on endothelial cells is increased by the hypoxia and low pH that is the classical tumor microenvironment [42]. It is necessary to verify these claims with more studies in the future.

The level of CXCL16 and CXCR6 are upregulated in tumor tissues and HCC cell lines. This high expression is associated with the increase of neutrophils in tumor tissues, and with a poor prognosis of HCC patients. In addition, triggering the CXCR6 signal pathway in HCC cells leads to the upregulation of IL-6 and IL-8. However, blocking the CXCL16-CXCR6 axis stops this effect. This suggests that the elevated expression of CXCR6 creates a protumor inflammatory environment, promoting HCC invasion [43].

#### 3.1.3. CX3CRn

CX3CL1 is the only member of the CX3C subclass. The CX3CL1/CX3CR1 axis contributes to the development of HCC. HCC patient tissues with a high level of both CX3CL1/CX3CR1 have a markedly lower proliferating cell nuclear antigen labeling index, which leads to a better prognosis in terms of overall survival and being disease-free, with fewer intra- and extrahepatic recurrences. These results indicate that CX3CR1 could be regarded as a prognostic factor [44]. CX3CR1 is also identified as an effective target for cancer immunoprevention [45]. It may regulate physiological levels and abnormal activity of inflammatory immune cells, helping to recover physical dynamic balance.

#### 3.1.4. CXRn

XCL1 is the sole member of the C subclass. X-C motif chemokine receptor1 (XCR1), as the only receptor of XCL1, suppresses liver cancer growth and tumorigenesis (at least in part) through the MAPK and PI3K/Akt signaling pathways. It does, however, promote metastasis via EMT [18].

### 3.2. E-Prostanoid Receptors

Prostaglandin E2 (PGE2) exerts its physiological effects by binding cognate prostanoid receptors, which are divided into EP1, EP2, EP3, and EP4. Normally, EP1-receptors bind to Gαq-proteins and trigger the signal pathway through PLC and enhanced intracellular Ca^2+^ concentrations. EP2 and EP4 receptors bind to G proteins, signaling via upregulating the level of intracellular cAMP and the activation of protein kinase A (PKA), while EP3 receptors bind to Gαi-proteins and signal by reducing intracellular cAMP. They may influence the growth, invasion, adhesion, metastasis, and angiogenesis of cancer cells, consequently participating in the tumorigenesis and progression of HCC [46].

The level of EP1 receptors is higher in HCC cells compared with normal human hepatocytes. PGE2 couples to the EP1 receptor, a process in which EGFR, Src, and p44/42 MAPK are all involved. Then, p44/42 MAPK activates the mTOR pathway, which in turn upregulates the YB-1 expression, promoting cancer cell invasion through regulating EMT-associated gene expression [47]. Also, the EP1 receptor has an impact on PGE2-mediated β1-integrin expression, which promotes HCC cell migration through the EP1/PKC/NF-κB/FoxC2/β1-integrin signal pathway [48]. Furthermore, EP1- and EP3-receptor antagonists markedly decrease cell proliferation and increase apoptosis in HCC cells [49].

The EP2 receptor has a vital influence on accelerating Huh-7 cell migration and invasion by triggering the EP2/Src/EGFR, an RTK, mediating the PI3K/Akt/mTOR pathway. mTOR upregulates the expression level of the Snail protein, thus regulating tumor cell proliferation, growth, and survival [50]. Meanwhile, the EP2 receptor and Bcl-2 are expressed at a high level, but the expression of Bax and cleaved caspase-3 is reduced in HepG2 and SMMC-7721 cells after stimulating with butaprost, a selective EP2-receptor agonist [51]. Consequently, EP2 may increase the proliferation and inhibit the apoptosis of HCC cells. Also, EP4 increases cell growth and invasive ability in human HCC cells via the Gs/AC/cAMP/PKA/CREB signaling pathway [46].

### 3.3. Lysophosphatidic Acid Receptors

Lysophosphatidic acid (LPA) is a biological lipid regulator with various physiological and pathological functions on different kinds of cells. Initially, LPA elicits its biological actions through coupling to three subclasses of the endothelial differentiation gene (Edg) family GPCR (LPAR1, LPAR2, and LPAR3). Apart from these receptors, there is another group from the Edg family, including LPAR4, LPAR5, and LPAR6. They regulate various cell biological functions such as motility, migration, and proliferation [52].

LPAR1, 3, and 6 mRNA and protein expression is high in the human hepatoma cell line HuH7. Stimulation with LPA promotes HuH7 proliferation and increased motility in dose-dependence [53]. LPA, HCC cells secreted in a paracrine mechanism, contributes to transdifferentiation of peritumoral tissue fibroblasts (PTFs) to a carcinoma-associated fibroblasts-like myofibroblastic phenotype. It upregulates specific genes related to a myo/contractile phenotype. After transdifferentiation, PTFs express α-smooth muscle actin and enhance proliferation, migration, and invasion of HCC [54]. Thus, blocking LPA is important for inhibiting transdifferentiation of myofibroblasts and HCC development.

LPAR1 protein is upregulated in HCC compared to normal tissues. Silencing LPAR1 markedly attenuates LPA-induced MMP-9 expression levels and the invasive ability of HCC cell. LPAR1 elevates MMP-9 expression via both PI3K/Akt and protein kinase Cδ (PKCδ)/p38 MAPK pathways [55]. In addition, LPAR1/LPAR3 is significantly elevated in the microenvironment. When knock-down LPAR1 or LPAR3, LPA-dependent cell migration happens via an LPAR3-Gi-ERK-pathway independent of LPAR1. This suggests that LPAR3 might mediate tumor invasiveness and expansion [56]. Moreover, LPA increases the expression of HIF-1α to induce VEGF expression [57]. Finally, the expression of LPAR6 also significantly increases in human liver tumors, but there is no sufficient basis for this mechanism [53].

### 3.4. Adrenergic Receptors

Adrenergic receptors (ARs) appear in a variety of tissues and mainly regulate the sympathetic nervous system (SNS) system and other cellular processes through coupling to catecholamines (norepinephrine, epinephrine). However, aberrant adrenergic signaling contributes to the initiation and deterioration of HCC, including cell motility and trafficking, angiogenesis, inflammation, activation of tumor-associated viruses, apoptosis/anoikis, cellular immune response, and EMT. The β2-, α1-, and α2-ARs are the primary subclass in the human liver. The β2-AR bundling with Gs and α2-AR bundling with Gi are related to the biochemical effector-AC-system, which catalyzes cyclic 3′,5′-adenosine monophsphate (cAMP) formation from ATP. Also, the α1-AR bind to Gq/11-protein and activate PLC [58].

α1-AR is lower in HCC tissues compared with those of nonadjacent, noncarcinoma livers. Tumor-induced stress also increases the catecholamine level upon chronic stimulation of the SNS that may result in desensitization and inhibition of the α1-AR-Gq/11-PLC-pathway [59]. However, β1- and β2-AR are the dominant subtypes in HCC tissues and cell lines. They stimulate the synthesis of cAMP from AC, which then activates PKA, eventually resulting in the phosphorylation of transcription factors. Activation of α1-AR and β2-AR results in the metalloprotease 7-dependent release of EGF-like ligands, which activate the EGFR and intracellular downstream signal, inducing HCC metastasis [60]. In addition, the β2-AR is overexpressed and highly correlated with poor prognosis in HCC patients after curative resection. Positive β2-AR protein expression is significantly associated with a high α-fetoprotein level, large tumor size, tumor encapsulation, vascular invasion, microsatellite formation, and poor differentiation. The β2-AR agonist promotes the growth of human cancer cells through activation of the cAMP/PKA, MAPK/ERK1/2, and PI3K/Akt signaling pathways [61,62]. It negatively regulates autophagy by interrupting the Beclin1/VPS34/Atg14 complex in an Akt-dependent fashion, resulting in HIF-1 stabilization, rearrangement of the HCC cells’ glucose metabolism, and the acquisition of resistance to sorafenib [63].

### 3.5. Angiotensin II Receptors

Angiotensin II (Ang II), a key bioactive peptide of the rennin-angiotensin system, mediates its biological effects via coupling to an angiotensin II type 1 receptor (AT1R) or AT2R. Although they belong to the same subclass of GPCRs, their distribution and intracellular signaling pathways are different. Both, however, have a crucial impact on neovascularization and tumor progression. For example, AT1R and AT2R have proangiogenic effects in colorectal cancer liver metastases and melanoma [64,65].

AT1 induces production of angiogenic factors such as VEGF, Tie-2, and angiopoietin-2 (Ang-2) in MHCC97H cells. Furthermore, VEGF and Ang-2 significantly increase blood vessel formation [66]. AT1R, which is expressed at inflammatory sites, assists AngII to produce angiogenic factors through AT1R/JAK2/STAT3/SOCS3 signaling pathway for vascular permeability and angiogenesis [67].

Some research suggests that AT2R has a proangiogenic function and works in cooperation with the AT1R subtype to upregulate the level of VEGF and angiogenic tube formation [68,69]. However, AT2R is thought to oppose the actions of the AT1R subtype in most pathophysiological environments and in some experimental angiogenic models [70]. Overexpression of AT2R inhibits cell vitality and promotes apoptosis through the cell death signaling pathway that depends upon inactivation of p42/44 MAPK (ERK1/2); activation of p38 MAPK, phosphorylase c-jun N-terminal kinase (p-JNK), caspase-8, and caspase-3; as well as regulation of CDK4 and cyclinD1 in SMMC7721 cells. It is interesting that a minor upregulation of AT2R shows the contrary effects in vivo, promoting cell proliferation and tumor growth in HCC [71]. These conflicting studies indicate that AT2R activation may have a double effect on HCC angiogenesis. Further research is required to understand the precise mechanisms of these phenomena.

### 3.6. Smoothened (Smo) Receptors

The Smo receptors, one of the class F GPCRs, are core components of the classical hedgehog (Hh) signaling pathway during embryogeny and adulthood [72]. It is well-established that the Hh pathway plays an essential role in the progression of HCC [73]. Further, Smo receptors are correlated with HCC recurrence by contributing to liver fibrosis and hepatocarcinogenesis [74,75,76].

Upregulation of the Smo proto-oncogene, and an enhancement in the stoichiometric ratio of Smo to patched (Ptc) mRNA levels, correlates with tumor size, a prognostic monitor in HCC biology [77]. It has been reported that the recipient polymorphism of Smo rs3824 has a comparatively lower recurrence-free survival rate, higher recurrence risk, and lower overall survival. This may be due to Smo rs3824 being located at the 3'-untranslated region (3‘-UTR), resulting in high activity of Hh pathway [78]. Furthermore, overexpression of Smo and MMP-9 are associated with accelerated migration and invasion in HCC tissues. Smo is regarded as a direct target of miR-338-3p. Use of miR-338-3p can disrupt liver cancer cell invasion via blocking Smo-mediated MMP-9 expression in SMMC-7721 cells [79]. Consequently, Smo receptors are considered to be correlated with HCC and HCC recurrence.

### 3.7. Orphan GPCRs

GPCRs comprise the largest family of transmembrane receptors, with more than 800 members, in which hundreds of members are classified as orphan GPCRs (oGPCRs), with no identifiable endogenous ligands [80]. Recently, some of these GPCRs have been related to HCC development and progression on the basis of their abnormal expression by various factors. For example, the high expression of the orphan G protein coupled receptor GPR49 is frequently observed in HCC with mutation in β-catenin. β-catenin promotes carcinogenesis through activation of the Wnt-signaling pathway, and several genes—including c-myc and cyclin D1—are the downstream targets in this pathway. Therefore, although much is still unknown, Gpr49 does seem to participate in HCC development [81].

GPR137 is elevated in some human HCC cell lines. Depletion of GPR137 via lentivirus mediated RNA interference (RNAi) in HCC cell lines HepG2 and Bel7404 remarkably reduces cell viability and colony-formation ability, which increases cell apoptosis [82]. However, GPR37 expression is lower in HCC compared to the adjacent nontumorous tissues. Furthermore, the transient GPR37 knockdown by siRNA in HuH7 cells strikingly decreases the apoptosis of hepatoma cells and increases cell proliferation by activating the PI3K-Akt signaling pathway [83]. Further research is required to elucidate the detailed molecular mechanisms of these receptors in the pathogenic mechanism of HCC in order to provide a basis for designing new drugs for HCC. Overall, studies have found that some GPCRs play an important role, as shown in Table 1.

## 4. GPCR-Based Treatments in HCC

HCC is the most stubborn cancer, resistant to chemotherapies for a variety of tumors, and there are too few drugs available for scientific chemotherapy at present. However, recent advances in the understanding of GPCRs and their downstream effectors reveal a large number of novel drug targets for groundbreaking strategies in HCC diagnosis, prevention, and treatment.

For example, blockade of CCL2/CCR2 signalling with a CCR2 antagonist, RDC018, inhibits HCC growth and metastasis, reduces postsurgical recurrence, and enhances survival. Furthermore, it suppresses murine liver tumor growth via inhibiting the recruitment of inflammatory monocytes and infiltration and M2-polarisation of tumorassociated macrophages (TAMs), resulting in a reversal of the immunosuppression status of the tumor microenvironment and activation of an antitumorous CD8^+^ T cell response [84]. In addition, mouse models of diet-induced HCC treated with a CCR5 antagonist maraviroc (MVC) generates lower a proliferation index, less liver fibrosis, lower levels of liver injury markers and chemokines, lower tumor burden than the model group, as well as higher survival rates [85]. This study suggests that MVC may serve as an effective treatment for HCC. Also, the N-terminal-methionylated RANTES (Met-RANTES), a selective CCR1 and CCR5 antagonist, has shown to be a helpful drug in the prevention and treatment of liver inflammation, fibrosis, and HCC in clinical development [24]. Furthermore, CXCR4 antagonist AMD3100 (plerixafor) synergistically suppress HCC progression when combined with antiangiogenic sorafenib [86,87]. This suggests a new molecule targeting CXCR4 for use as a potential therapeutic agent to prevent the HCC progression and spreading. Also, CCX771—a selective CXCR7 antagonist—significantly decreases CXCR7-mediated cell invasive and proliferative ability through the activation of MAPK pathway proteins and proangiogenic signaling pathways [40]. Consequently, targeting CXCR7 may inhibit migration and present an appropriate target molecule for HCC therapeutic strategy.

A crosstalk between the PGE2 and EGFR/c-Met signaling pathways affects HCC cell invasive ability. PGE2-induced transactivation of EGFR in HCC cells occurs through activation of c-Src, followed by c-Met activation. Thus, when treating with the EP1 receptor antagonist ONO-8711, the PGE2-mediated phosphorylation of EGFR and cell invasion are inhibited [88,89]. Furthermore, the EP1-receptor antagonist AH6809 reduces the viability of HCC cells in HuH-6 and HuH-7 cells. Moreover, treatment with a combination of AH6809 and COX blockers (SC-560 and NS-398) shows a more dramatic inhibition of cell proliferation than the single drug alone. This demonstrates that the EP1 receptor has a vital potential for HCC treatment [90]. Furthermore, inhibition of HSC-derived PGE2 with an EP4 antagonist(AH23848) could disturb HSC-mediated MDSC accumulation and HCC growth in vivo. Thus, EP4 may be a reasonable target protein for therapeutic use in HCC [91].

The β2-AR antagonist propranolol has an impact on enhancing sorafenib efficacy, since blocking β2-AR signaling results in HIF-1α autophagic degradation [63].

Furthermore, the endothelin type A receptor (ET_A_R) selective inhibitor BQ-123 suppresses ET-1-induced migration and invasion, and downregulates MMP-3 production in HepG2 cells via inhibiting activation of the ERK1/2 and Akt pathways [92]. This shows that ET_A_R could be a potential target for HCC treatment.

GDC-0449 (vismodegib), an antagonist of Smo, significantly decreases tumor size and cell infiltration of HCC in HCC-bearing mice. Gene expression of the sonic hedgehog (Shh) pathway molecules is altered, such as the upregulated Shh expression and downregulated smoothened expression in tumor areas after treatment with GDC-0449 [93]. These GPCR-based treatments are shown in Table 2.

## 5. Conclusions

Although GPCRs constitute the largest superfamily of transmembrane receptors participating in signal transmission, in clinical practice, only a few anticancer drugs have been applied to HCC. In addition, the function played by GPCRs and its cognated ligands in HCC development and pathophysiological progression is intricate. Therefore, GPCRs and their downstream-regulated effectors provide many drug targets for novel strategies in HCC prevention and treatment. Consequently, the full knowledge of the transduction crosstalk connecting several GPCR-dependent signals with other transduction pathways will promote further research regarding the biological potential of these receptors, meanwhile providing valuable avenues for the development of novel, GPCR-based therapeutic strategies for cancer diagnosis. Finally, the persistent efforts to fully describe a rich source of GPCRs will certainly lead in the near future to the identification of novel targets toward groundbreaking pharmacological treatment strategies in HCC patients.

## Figures and Tables

**Table 1:** Role of GPCRs in HCC.

GPCRs	Protein Expression	Cognate Ligands	Role in HCC	Biological Model
CCR1	Up	CCL2, CCL3, CCL5	Increased migration [21], increased angiogenesis [19,20], increased invasion [15]	HCC patients, DEN-induced mice model, myelo-ablated syngeneic HBs antigen transgenic mice, CCR1-deficient mice, human HCC cell line (HCCLM3)
CCR2	Up	CCL2	Increased metastasis [22], increased angiogenesis [23]	HCC patients, CD11b/Gr1mid cells, transgenic CD11b-diphtheria toxin receptor mouse model, CCR2- deficient mice
CCR5	Up	CCL3	Alter tumor microenvironment: increased IL-6 and TNF-α [24]	HCC patients, Mdr2-knockout mice
CCR6	Up	CCL20	Increased migration [25], increased invasion, migration and angiogenesis [26]	HCC patients, Tregs cell, human HCC cell line (HepG2, MCF-7)
CCR7	Up	CCL21, CCL19	Increased angiogenesis [27], alter tumor microenvironment: increased IL-10 and TGF-β1, decreased level of IL-12 and IFN-γ [28]	HCC patients, DEN-induced mice model
CCR9	Up	CCL25	Increased proliferation [29]	HCC patients, human HCC cell lines (HepG2, Huh7, Hepan3B)
CXCR2	Up	CXCL5	Increased EMT [16], increased migration and invasion [16,17,30], increased angiogenesis [31]	HCC patients, xenograft mouse models, orthotropic nude mice, human HCC cell lines (MHCC97-L, MHCC97-H, HCCLM3, HepG2, Huh7)
CXCR3	Up	CXCL9, CXCL10	Increased invasion and migration [32]	HCC patients, metastasis mice model, human HCC cell lines (Hep3B, PLC/PRF/5, Huh7)
CXCR4	Up	CXCL12	Increased invasion and migration [33,35,36,38], increased EMT [34,36,37,38]	HCC patients, human HCC cell lines (Huh7, Hep3B, MHCC97, SMMC7721), hepatic stellate cell (LX-2)
CXCR6	Up	CXCL16	Increased invasion, alter tumor microenvironment: increased proinflammatory cytokine production such as IL-17, IL-6, and IL-8 [43]	HCC patients, xenograft mouse models, human HCC cell lines (Hep3B, HepG2, Huh7, PLC/PRF/5, SK-HEP-1, MHCC97L, MHCC97H, HCCLM3), human liver cell line (L-02)
CXCR7	Up	CXCL12	Increased proliferation, migration and invasion [39,40,41], increased angiogenesis and EMT [42]	HCC patients, DEN-induced mice model, human micro-vascular endothelial cell line (HMEC-1)
CX3CR1	Down	CX3CL1	Immunoprevention [44,45]	HCC patients
XCR1	Down	XCL1	Suppresses liver cancer growth and tumorigenesis, increased metastasis [18]	human HCC cell lines (Huh-7, HepG2, Hep3B, SMMC7721, HCLM3, HCCLM6, MHCC-97L, MHCC-97H)
EP1	Up	PGE_2_	Increased invasion [47], increased migration [48], increased apoptosis [49]	HCC patients, human HCC cell lines (Huh-7, Hep3B, HEK293 cells)
EP2	Up	PGE_2_	Decrease apoptosis [50,51]	Human HCC cell lines (SMMC-7721, HepG2)
EP4	Up	PGE_2_	Increased proliferation and invasion [46]	Human HCC cell line (Huh-7)
LPAR1	Up	LPA	Increased invasion and migration [55], increased proliferation [56]	HCC patients, human HCC cell lines (SK-Hep1, HepG2 and HuH-7)
LPAR3	Up	LPA	Increased invasion and migration [56]	HCC patients, human HCC cell lines (SK-Hep1, HepG2 and HuH-7)
LPAR6	Up	LPA	Increased proliferation, invasion and migration [53,56]	HCC patients, subcutaneous implantation HCC female CD-1 nude athymic mice, human HCC cell lines (HepG2, HuH-7 PLC/PRF/5, and HLE)
α_1_-AR	Down	Catecholamines	Increased ALT, alter tumor microenvironment: decrease energy expenditure, low levels of thyroid hormones, induce hepatocyte injury [59]	HCC patients
β_1_-AR	Up	Catecholamines	Increased proliferation [60]	HCC patients
β_2_-AR	Up	Catecholamines	Increased proliferation, invasion and migration [61,62,63]	HCC patients, DEN-induced HCC mice model, xenograft male nude mice model, human HCC cell lines (SMMC-7721, HepG2, MHCC-97L) human normal liver cell line (L-02)
AT_1_R	Up	Ang II	Increased angiogenesis [67]	human HCC cell lines (MHCC97-L, Bel-7402)
AT_2_R	Up or down	Ang II	Increased proliferation [68], increased angiogenesis [69,70,71]	Intrahepatic tumor mice model, wild-type and AT2-deleted mice, human HCC cell lines (SMMC-7721, Bel7402, HepG2), human fetal liver cell line (L-02)
Smo	Up	Sonic hedgehog	Increased invasion and migration [77,78,79,80]	HCC patients, normal human hepatic cell (L-02), human liver cancer cell lines (PLC/PRF/5, Hep3B, SK-HEP-1, Huh7, Bel-7402, and SMMC-7721)
GPR49	Up	Unknown	Increased proliferation [81]	HCC patients, human HCC cell lines (PLC/PRF/5 and HepG2)
GPR137	Up	Unknown	Increased proliferation, decreased apoptosis [82]	Human HCC cell lines (HepG2, Bel7402, Bel7404, SK-HEP-1, Hep3B, SMMC-7721), human embryonic kidney cell line (HEK293T)
GPR37	Up	Unknown	Increased proliferation, decreased apoptosis [83]	HCC patients, human HCC cell line (Huh-7)

**Table 2:** GPCR-based treatments in HCC

Antagonists	Targeted-GPCR	Mechanism	Biological Model	Reference
RDC018	CCR2	Inhibits the recruitment of inflammatory monocytes, infiltration, and M2-polarisation of tumor-associated macrophages resulting in reversal of the immunosuppression status of the tumor microenvironment and activation of an antitumorous CD8^+^ T cell response.	HCC patients, orthotopic HCC model, murine HCC cell line (Hepa1-6, LPC-H12, and H22), human HCC cell lines (Hep3B, HepG2, BEL-7404, SMMC-7721, Huh-7, PVTT-1, MHCC-97L, MHCC-97H, and MHCC-LM3), human liver cell lines (HL-7702 and L-02)	[84]
Maraviroc	CCR5	Inhibits hepatic stellate cells activation markers such as phosphorylation of p38 and ERK, and increases hepatocyte survival.	Choline-containing diet male C57BL/6 mice	[24,85]
Met-RANTEs	CCR1, CCR5	Reduced inflammation by reducing periductal accumulation of CD24^+^ oval cells and abrogation of fibrosis.	Wild-type C57Bl/6J, CCR5-deficient mice, double mutant Mdr2:CCR5 and Mdr2:CCR1 DKO mice	[24]
AMD3100	CXCR4	In combination with sorafenib treatment block CXCR4/SDF1α, prevents the infiltration of tumor-associated macrophages. enhanced antiangiogenic effect and suppress local and distant tumor growth in HCC.	Orthotopically implanted HCC Male C3H/HeNCrNarl mice, murine HCC cell line(HCA-1), human HCC cell line(JHH-7)	[86,87]
CCX771	CXCR7	Inhibited CXCR7-induced phosphorylation of ERK1/2 signaling and secretion of the proangiogenic factors VEGF-A.	HCC patients, hepatic cell lines (L-02 and QSG7701), human HCC cell lines (QGY7703, HepG2, Hep3B, MHCC97L, MHCC97H, HCCLM3, HCCLM6)	[40]
ONO-8711	EP1	Decreased EGFR phosphorylation and tumor cell invasion.	HCC patients, human HCC cell lines (Hep3B, Huh7, and HepG2)	[88,89]
AH6809	EP1	Combination of EP1 receptor antagonist and COX inhibitors produced a significantly greater cell growth inhibition than the single agent alone.	HCC patients with hepatitis virus-associated chronic liver disease, human HCC cell lines (HuH-6 and Huh7)	[90]
AH23848	EP4	Inhibition of HSC-derived PGE_2_ could inhibit HSC-induced MDSC accumulation and HCC growth.	Adult male BALB/c mice injected intra-hepatically with cell suspension, mouse hepatoma cell line (H22)	[91]
Propranolol	β_2_-AR	Enhanced autophagy, HIF-1α destabilization, tumor growth suppression, and improved anti-tumor activity of sorafenib.	Human patients, DEN-induced HCC mouse model, human HCC cell line (SMMC-7721)	[63]
BQ123	ET_A_R	Blockade of ET_A_R inhibits HCC cell migration and invasion via blocking ERK1/2 and Akt signaling pathways and lowering MMP-3 expression.	Human liver cell line (L-02), human HCC cell lines (MHCC97-L, Bel-7402, SMMC-7721, and HepG2)	[92]
GDC-0449 (vismodegib)	Smo	Blockade of Smo and Shh signaling pathway result in a successful mitigation of ML-1 growth.	Implantation of mice hepatoma ML-1 cells C57BL/6 mice model	[93]

## Abbreviations

GPCRs	G protein-coupled receptors
HCC	hepatocellular carcinoma
GRKs	G protein receptor kinases
AC	adenylyl cyclases
GABA_B_	γ-aminobutyric acid type B
GDP	guanosine diphosphate
GTP	guanosine triphosphate
DCs	dendritic cells
IL-12	interleukin-12
IFN-γ	interferon-γ
3‘-UTR	3'-untranslated region
p-JNK	phosphorylase c-jun N-terminal kinase
PLC	phospholipase C
TGF-β	transforming growth factor-β
MAPK	mitogen-activated protein kinase
DEN	*N*-nitrosodiethylamine
HBs	hepatitis B virus surface
MMPs	matrix metalloproteinases
RNAi	RNA interference
EMT	epithelial-mesenchymal transition
EGFR	epidermal growth factor receptor
PDGFR	platelet-derived growth factor receptor
IGF1R	insulin-like growth factor 1 receptor
siRNAs	small interfering RNAs
RTKs	receptor tyrosine kinases
VEGF	vascular endothelial growth factor
PGE2	prostaglandin E2
PKA	protein kinase A
YB-1	Y-box-binding protein 1
LPA	lysophosphatidic acid
Edg	endothelial differentiation gene
PTFs	peritumoral tissue fibroblasts
PI3K	phosphoinositide 3-kinase
PKCδ	protein kinase Cδ
HIF-1	hypoxia-inducible factor-1
ET_A_R	endothelin type A receptor
ARs	adrenergic receptors
SNS	sympathetic nervous system
cAMP	cyclic 3′,5′-adenosine monophsphate
CREB	cAMP response element binding protein
Ang II	angiotensin II
AT1	angiotensin II type 1
Ang-2	angiopoietin-2
Hh	hedgehog
Ptc	patched
oGPCRs	orphan GPCRs
TAMs	tumor associated macrophages
Met-RANTES	methionylated RANTES
Shh	sonic hedgehog
